# Underlying risk factors and their relationship with extent of coronary vessel involvement in patients undergoing coronary angiography in North of Iran

**DOI:** 10.22088/cjim.9.4.361

**Published:** 2018

**Authors:** Fahimeh Kazemian, Seyed Farzad Jalali, Karimollah Hajian-Tilaki, Afsaneh Arzani, Kamyar Amin

**Affiliations:** 1 Non-Communicable Pediatric Diseases Research Centers Health Research Insitute, Babol University of Medical Sciences, Babol, Iran; 2 Department of Cardiology, Clinical Research Development unit of Ayatollah Rouhani Hospital, Babol University of Medical Sciences, Babol, Iran; 3 Social Determinants of Health Research Center, Health Research Institute, Babol University of Medical Sciences, Babol, Iran; 4 Nursing Care Research Center, Health Research Institute, Babol University of Medical Sciences, Babol, Iran; 5 Nursing & Midwifery Faculty, Babol University of Medical Sciences, Babol, Iran

**Keywords:** Risk factors, Coronary Angiography, Coronary Vessels, Coronary Stenosis

## Abstract

**Background:**

Coronary artery disease (CAD) is one of the most progressive and life-threatening diseases and is the first leading cause of death affecting both genders in Iran. The present study aimed to determine the extent of coronary vessel involvement and relevant relationship with several underlying risk factors.

**Methods:**

In this cross-sectional study, 1452 patients undergoing angiography who met the inclusion criteria were recruited consecutively in Babol, Iran during 2016. Data collection was performed through a questionnaire including demographic and clinical characteristics and information on underlying diseases via an interview with the patient and looking into the patients’ records.

**Results:**

Of these patients, 459 (31.6%) had normal coronary arteries, 220 (15.1%) suffered from non-obstructive CAD and 773 (53.3%) had one, two or three-vessel obstructive involvement. The results of multiple logistic regression showed that the chances of having coronary artery involvement in patients with diabetes (OR=1.55, p=0.012), age> 60 years old (OR=3.52, P=0.001), male gender (OR=2.54, P=0.001), history of heart attack (OR=3.16, P=0.001), and history of hospitalization because of cardiac problem (OR=1.51, P=0.021) significantly increased.

**Conclusions:**

Diabetes, male gender, age over 60 years old, history of myocardial infarction and history of hospitalization due to cardiac problem were related to the extent of coronary vessels involvement. Therefore, it is recommended to practice preventive measures more extensively in this regard.

Coronary artery disease (CAD) is one of the most common chronic, progressive and life-threatening diseases around the world (1,2). It is the cause of one third of mortality rates in the world (2). It imposes huge economic losses on the society more than any other diseases (1). This is also the major cause of disability in the patients (3). The prevalence of this disease is increasing in the developing countries rapidly so that in recent years, 78% mortality rate has been attributed to CAD in these countries (2, 3). In Iran, according to statistics, 46% mortality rate because of cardiovascular diseases was reported, that is the first leading cause of death in both genders (4,5). In the past, the disease was more common in the elderly, but nowadays young people are also facing it (6). The clinical spectrum of coronary heart disease varies from asymptomatic ischemia to chronic stable angina, unstable angina, acute myocardial infarction, ischemic cardiomyopathy, sudden cardiac death, arrhythmias and cardiogenic shock (7).

Also, the disease is divided into two groups of obstructive (stenosis more than 50% of diameter of the vessel) and non-obstructive (stenosis less than 50% of diameter of the vessel) (8). Although coronary artery disease is one of the major health care problems in modern societies, by determination, control of risk factors, correct diagnosis and treatment can prevent many deaths caused by it (9). There are many underlying risk factors for this disease including male sexuality, smoking or being near smoking people, overweight and obesity, inappropriate diet, high blood pressure and hypertension, diabetes, hormone therapy, psychological factors such as depression and stress (10-12). Many studies have shown the relationship between some of these underlying factors with the severity of coronary artery involvement with different results in this regard (6- 7, 11). The abnormal changes in the ECG and positive CRP can be an underlying risk factor for the disease (13). Multiple invasive and non-invasive methods are used to determine the severity and extent of CAD but ultimately, angiography is the gold standard for a definitive diagnosis (5, 14).

Due to increasing prevalence of atherosclerotic CAD, coronary artery angiography and percutaneous coronary interventions are used extensively as standard methods for examining coronary anatomy and treating atherosclerotic lesions over the last few decades (15). Because of the high-demand for angiography at the cardiac center in Babol, and extensive literature review of relevant papers by the authors showed that no knowledge existed in the relation of the number of involved coronary vessels and its association with underlying risk factors in patients undergoing angiography. In regard to the importance of the topic, the present study aimed to determine the extent of involved coronary vessels with its association to some underlying risk factors in Babol, North of Iran in 2016.

## Methods

This cross-sectional study was conducted on 1452 patients who underwent angiography at Ayatollah Rouhani Teaching Hospital in Babol during 2016. All patients who required angiography that referred from the clinics of cardiologist to Babol angiography center were entered into this study by census. Participants with history of angiography or angioplasty, chronic cardiac failure, congenital heart disease, history of cardiac surgery, aged less than 18, loss of consciousness or inability to communicate verbally were excluded from this study. The present study was approved by the Ethics Committee of Babol University of Medical Sciences (No. 306446) and all patients had given an informed written consent and ethical issues on the confidentiality of information the patients were considered. Demographic and clinical characteristics were age, gender, place of residence, education, history of smoking, smoking spouse, addiction, hypertension, hyperglycemia, family medical history hospitalization history owing to cardiovascular diseases, history of myocardial infarction and stroke and the severity of coronary artery involvement.

Data collection was performed through a questionnaire in two parts. In the first step, demographic data and clinical information on underlying diseases were collected through an interview with the patient and patients’ records prior to angiography. 

In the second part of questionnaire, the results of angiography after doing angiography by the cardiologist were documented from angiographic report papers. Angiography results encompassed: normal state, non-obstructive involvement (less than 50%) and one, two, and three-vessel coronary artery stenosis (more than 50% involvement). Number of vessel and extent of involvement were our primary outcome.

In the statistical analysis, we used SPSS Version 14. Categorical data was analyzed using chi-square statistical test for quantitative data in our comparison. Multivariate logistic regression was applied to estimate the adjusted odds ratio (OR) of risk factor on the extended at least one vessel coronary involvement. The effect of other risk factors as covariate had been adjusted. Confidence interval in this study was 95% and significant level was less than 0.05. 

## Results

Of the 1452 patients understudy, 663 (45.6%) were males and 789 (54.4%) were females. The mean and standard deviation of the age of the patients were 57.8±11 years. Among all the patients, 459 (31.6%) individuals had normal coronary artery, 220 (15.1%) patients suffered from non-obstructive involvement and 773 (53.3%) had obstructive involvement of 1, 2 or 3 vessels. According to chi-square test, patients aged 41-60 years needed angiography more than others and the most extensive coronary artery involvement (obstruction in three vessels) was observed in those aged over 60 years (table 1). 

**Table 1:** Distribution of the participants, based on the extent of involvement and its relationship with gender and age

** Extent of ** ** involvemen** **t** **Varibles **		**Normal** **N(%)**	**non-obstructive involvement** **N(%)**	**Obstruction**			**Tota** **l**	**P-value**
				**vessel involved** **N(%)**	**2 vessels involved** **N(%)**	**3 vessels involved** **N(%)**		
15 (17.5)	Age groups	48(55.8)	15 (17.5)	12 (14)	6 (6.9)	5 (8.5)	86	0.001
135(17.15)		272 (34.8)	135(17.15)	129 (16.4)	129 (16.4)	120(15.5)	787	
70 (12.1)		137 (23.7)	70 (12.1)	91(15.1)	126 (21.8)	155 (26.8)	579	
220 (15.1)		459 (13.6)	220 (15.1)	232 (15.9)	261 (17.9)	280 (19.2)	1452	
72 (10.8)	Sex	176 (26.5)	72 (10.8)	113 (7.8)	141 (9.7)	16 (19.1)	663	0.001
148 (18.7)		28 (35)	148 (18.7)	119 (8.2)	120 (8.3)	119 (8.2)	789	

There was no significant difference between the mean age of men and women who required angiography (p=0.6). There was a significant difference between men and women, in terms of the results of the CAD (p<0.001). The prevalence of hypertension (36% vs. 58%), hyperlipidemia (39% vs. 56%) and diabetes (23/8% vs. 39%) was higher in women and history of heart attacks (17.3% vs. 9.8%) was higher in men (p<0.001).

We used chi-square test to compare two groups with CAD (stenosis< 50% and stenosis> 50%) in terms of some variables. History of heart attack (5% vs. 18.7%, p<0.001), history of hospitalization due to cardiac problems (27.2% vs. 41.7%, p<0.001) and smoking (10% vs. 16.7%, p=0.01) were significantly higher in the second group (stenosis> 50%) than the first group; however, the variable of having a smoker spouse (22% vs. 12.5%, p<0.001) in the first group (stenosis< 50%) was significantly higher than the second group. The patients in the stenosis> 50% group were also placed into three groups in terms of the number of involved vessels (1, 2 or 3 vessels) and were compared in terms of associated risk factors. The difference between the three groups was significant only in terms of the variable of history of heart attack (p=0.04). There was no significant difference between the three groups in terms of the mean duration of hypertension and hyperlipidemia, duration of smoking and addiction; however, there were significant differences between the groups in terms of duration of diabetes (p=0.04). There was no significant difference between the urban and rural patients in terms of the number of involved vessels (p=0.3). Table 2 indicated significant relationships between variables of hypertension, diabetes, smoking, addiction, having a smoker spouse, history of heart attack, hospitalization history due to cardiac disease with the extent of coronary artery involvement (p<0.05).

**Table 2:** Distribution of the participants, based on the underlying factors and their association with the results of angiography

** Extent of involvement**	**Without involvement** **N(%)**	**Nonobstructive stenosis** **N(%)**	**Obstructive stenosis**			**Total** **N(%)**	**P-value**
**Underlying Factors**			**1 Vessel** **N(%)**	**2 Vessels** **N(%)**	**3 Vessels** **N(%)**		
Hypertension	200 (28.7)	108 (15.5)	112 (16)	133 (19)	143 (20.5)	696 (48)	0.02
Hyperlipidemia	201 (28)	119 (17)	126 (17.9)	129 (18.3)	129 (18.3)	704 (48.4)	0.4
Diabetes mellitus	110 (23.5)	70 (15)	86 (18.4)	84 (18)	117 (25)	467 (34)	0.001
Smoking	36 (19.2)	11.7) 22)	31 (16.5)	47 (25)	51 (27.2)	187 (12.8)	0.001
Smoker spouse	71 (32.8)	48 (22.2)	34 (15.7)	28 (13)	35 (16.2)	216 (14.8)	0.04
Addiction	21 (18.2)	13 (11.3)	14.7) 17)	29 (25.2)	35 (30.4)	115 (8)	0.001
Heart attack history	37 (19)	11 (5.7)	31 (16)	28) 54)	31) 60)	193 (13.2)	0.001
History of stroke	18 (27.6)	12.3) 8)	13 (20)	18.4) 12)	21.5) 14)	65 (4.5)	0.4
History of heart hospitalization	126 (24.7)	11.8) 60)	86 (16.9)	21.6) 110)	25) 127)	509 (35)	0.001
Family history of heart disease	191 (31.1)	17) 104)	87 (14.1)	105 (17.1)	127 (20.6)	614 (42.2)	0.75

In the final analysis, the variables were inserted into the multiple logistic regression models using backward method. As table 3 shows, factors such as age over 40, hypertension, diabetes, gender, smoking, hospitalization due to cardiac problems and history of heart attack were the influential variables. Although, variables of hypertension and smoking had no significant effect on increasing the chances of obstructive involvement compared to non-obstructive involvement (the OR and p-value of the above variables are shown in table 3).

**Table 3:** Adjusted odds ratio of the underlying factors of coronary artery disease, (95% confidence interval in multiple logistic regression analysis)

**Variables**		**OR** (1) **( 95% CI)** **N=1452**	**P value**	**OR ** (2) ** (95% CI)** **N=993**	**P value** (3)
positive negative	Hypertension	1.23 (0.97-1.56) Ref	0.092	Ref	---
positive negative	Diabetes mellitus	1.86 (1.42-2.42) Ref	0.001	1.55 (1.10-2.18) Ref	0.012
positive negative	Smoking	1.99 (1.30-3.04) Ref	0.001	---------	-----
male female	Sex	1.52 (1.17-1.96) Ref	0.001	2.54 (1.83-3.54) Ref	0.001
positive negative	Heart attack history	1.55 (1.04-2.32) Ref	0.031	3.16 (1.643-6.097) Ref	0.001
positive negative	History of heart hospitalization	1.39 (1.08-1.81) Ref	0.012	1.51 (1.06-2.15) Ref	0.021
41-60 y < 40 y	Age	2.32 (1.44-3.67) Ref	0.001	1.81 (0.88-3.89) Ref	0.103
> 60 y < 40 y		3.98 (2.44-6.67) Ref	0.001	3.52 (1.69-7.33) Ref	0.001

(1) Adjusted odds ratio of the underlying factors of coronary artery disease vs. no coronary artery disease

(2) Adjusted odds ratio of the underlying factors of obstructive involvement vs. Nonobstructive involvement

(3) wald statistics using logistic regression

## Discussion

Based on the findings of this study, there were significant relationships between the extent of coronary artery involvement with some underlying risk factors such as age, male gender, diabetes, smoking, history of heart attack and history of hospitalization due to cardiac problem and duration of diabetes. The results of this study showed that there was no significant relationship between the extent of involvement of coronary and variables of hyperlipidemia and history of stroke. In line with the findings of our study, Masoumi and Nasri et al. found that there was no significant relationship between the extent of coronary artery involvement and hyperlipidemia (16). Similar to the results of the present study, Golmohammadi et al. found that there was significant relationship between the number of involved coronary arteries with the variables of age and history of heart attack. But contrary to the results of our study no significant difference was observed in term of gender (17). It is worth mentioning that the number of samples in our research has been almost five times the size of the above study.

In this study, patients with cardiac history and those with hospitalization history because of cardiac problem, the probability of developing coronary artery involvement were 1.5 and 1.3 times more than the normal people, respectively. One-vessel and three-vessel involvement were the lowest and highest prevalent types of involvement in our finding which was similar to the findings of other studies (17- 18). Nonetheless, our results are not consistent with the findings of Calvet and Hosseini et al.’s studies (19-20). Nough et al. showed that two-vessel involvement was the most common type of involvement; nevertheless, it was the least common type in other studies (15- 16, 21). These differences can be attributed to the sample size, data collection method, time period and geographical region.

In another study among older patients showed that one-vessel stenosis was more common among women and on the other hand, two and three-vessel stenosis were more common in men and this is similar to the findings of the present study (20). Whereas in another study showed that all types of involvement (one, two and three-vessel involvements) were significantly higher in men, which is to some extent consistent with the results of our study. In the present study, men were mostly suffering from cases of stenosis> 50%; while, women were mostly suffering from stenosis< 50% cases. Unlike these results, in a study conducted by Mohseni et al., no significant difference was observed between the two groups in terms of gender (22). Inconsistent with the result of the present study, Parsa et al. showed that there was no significant relationship between the two groups in terms of smoking (23). Despite the fact that smoking is a major risk factor for cardiovascular diseases (24); Aygul et al. argue that hypertension and diabetes are significantly less common among smokers and this is similar to the findings of the present study (25).

A similarity to our finding, in previous studies also the mean age of patients undergoing angiography was reported to be approximately 55-60 years (16, 21, 26). Unlike other studies, we did not find a significance difference between men and women in terms of their mean age (13, 21). Moreover in patients over the age of 60, the probability of obstructive involvement was three times larger than that of non-obstructive involvement. Previous studies have also shown that the severity of coronary artery involvement significantly increases over time (17, 20).

In the curent study, most of the individuals admitted to Babol angiography center were men, which is similar to most studies conducted in different Iranian cities (7, 16, 20- 21,26).The results of other studies show that women had significantly higher normal angiography cases than men (27- 28). In this regard, Khalili et al. studied a population of Iranian men and women over 10 years and reported that the prevalence of CAD in men is almost twice that of women (29). In the present study, the prevalence of coronary artery involvement was 1.5 times higher in men; while in Mohammad’s study, men were 3.4 times more likely to experience coronary artery involvement (6). The findings of the written study showed that the frequency of risk factors was significantly lower in those with normal angiography than that of the patients. In the study of Bidel et al., there was no significant difference between normal people and patients, in terms of high hyperlipidemia levels, which contradicts the result of the present study (24).

This study found that the prevalence of risk factors of diabetes, hyperlipidemia and hypertension was significantly higher in women than in men. Surprisingly, despite the lower prevalence of these factors in men, normal angiography was more prevalent in women than men. Unlike the foregoing findings, another study showed that diabetes and hyperlipidemia were more prevalent in men than in women but there was no significant difference between the two groups in terms of hypertension(7).

Similar to the findings of the present study, the multivariate logistic regression model used in the study of Bidel et al. showed that the probability of developing coronary artery involvement is directly correlated with the variables of smoking, hypertension, male gender and a history of cardiovascular diseases in the family. Nonetheless, their study showed that hyperlipidemia was associated with an increased risk of coronary involvement, which is not consistent with the present study (24). We found in our study, the probability of developing obstructive coronary artery involvement in diabetic patients was 1.5 times higher than that of non-obstructive involvement. In this regard, Masoumi (16) and Nasri et al. in their study showed that in diabetic patients, the risk of two or three-vessel involvement was twice more than that of one-vessel involvement; while, this ratio was 1.2 in the study of Hosseini et al. (30).

The findings of this study include some limitations. It is worth noting that since some of the data in this study are based on a self-report approach, they may be sensitive to biases such as patient claim. Moreover, psychosocial factors may prevent patients from answering honestly to some questions about addiction. This study design is a cross-sectional in nature, thus it must be a caution in interpreting of causal association of risk factors with visual involvement. Whaterer way, one of the strengths of this study was large sample size which represents higher reliability of our results. On the other hand, this study investigated the relationship between the extent of coronary artery involvement with the duration of the risk factors such as hypertension, hyperlipidemia, diabetes, as well as the duration of smoking and addiction. These relationships have not been addressed in previous studies.

 In conclusion, our study showed that diabetes, smoking, history of heart hospitalization were correlated with the CAD; but, the age of over 60, male gender, history of heart attack with more than twice chance of obstructive involvement. Therefore, authorities are recommended to hold effective training courses on preventive measures in this area.
